# Reverse transcription strand invasion based amplification (RT-SIBA): a method for rapid detection of influenza A and B

**DOI:** 10.1007/s00253-016-7491-y

**Published:** 2016-04-11

**Authors:** Kevin Eboigbodin, Sanna Filén, Tuomas Ojalehto, Mirko Brummer, Sonja Elf, Kirsi Pousi, Mark Hoser

**Affiliations:** 1 Research and Development, Orion Diagnostica Oy, P. O. BOX 83, FI-02101 Espoo, Finland; 2 Molecular Biology, GeneForm Technologies, Broadstairs, UK

**Keywords:** Influenza, RNA, Virus, Amplification, Isothermal, Diagnostics

## Abstract

Rapid and accurate diagnosis of influenza viruses plays an important role in infection control, as well as in preventing the misuse of antibiotics. Isothermal nucleic acid amplification methods offer significant advantages over the polymerase chain reaction (PCR), since they are more rapid and do not require the sophisticated instruments needed for thermal cycling. We previously described a novel isothermal nucleic acid amplification method, ‘Strand Invasion Based Amplification’ (SIBA®), with high analytical sensitivity and specificity, for the detection of DNA. In this study, we describe the development of a variant of the SIBA method, namely, reverse transcription SIBA (RT-SIBA), for the rapid detection of viral RNA targets. The RT-SIBA method includes a reverse transcriptase enzyme that allows one-step reverse transcription of RNA to complementary DNA (cDNA) and simultaneous amplification and detection of the cDNA by SIBA under isothermal reaction conditions. The RT-SIBA method was found to be more sensitive than PCR for the detection of influenza A and B and could detect 100 copies of influenza RNA within 15 min. The development of RT-SIBA will enable rapid and accurate diagnosis of viral RNA targets within point-of-care or central laboratory settings.

**Electronic supplementary material:**

The online version of this article (doi:10.1007/s00253-016-7491-y) contains supplementary material, which is available to authorized users.

## Introduction

Influenza A and B are among the most common causes of acute human respiratory illness and are associated with high morbidity and mortality rates in infants, the elderly, and immunocompromised individuals (Mallia and Johnston 2007; Simonsen et al. 2000). Today, patients with influenza infection may be treated with antiviral drugs; however, these treatments are only effective when started within the first 2 days of the illness (Stiver 2003). Thus, timely and accurate diagnosis of influenza plays an important role in targeting antiviral treatment and, furthermore, has a significant role in the prevention of the misuse of antibiotics, since the symptoms of influenza are often similar to those of other respiratory illnesses caused by bacterial infections (Low 2008).

Nucleic acid amplification tests (NAATs) are becoming the method of choice for routine diagnosis of influenza and other respiratory viruses within clinical laboratory settings. NAATs offer superior sensitivity and specificity over serology, immunoassays, and traditional viral culture-based detection methods (Mahony et al. 2011). Real-time reverse transcription polymerase chain reaction (RT-PCR) assays have been developed for the diagnosis of influenza and remain the most common platform used in NAAT. The RT-PCR method includes, as a first incubation step, the reverse transcription of RNA to complementary DNA (cDNA) by a transcriptase enzyme, followed by a PCR cycling stage. A RT-PCR run is typically completed within 2 h. Most reverse transcriptases are not thermostable; consequently, the reverse transcription step must be performed prior to the PCR step (Gerard et al. 2002). Typically, RT-PCR requires the use of heavy and sophisticated thermal cyclers and skilled personnel, which limits its use for in field or point-of-care applications. In an attempt to address this drawback, isothermal nucleic acid amplification platforms have been developed, which do not require the use of heavy, sophisticated thermal cyclers. Instead, isothermal nucleic acid amplification methods require only small instruments, enabling NAATs to be performed within point-of-care settings. This has the potential to allow prompt treatment of patients and limit the inappropriate use of antibiotics and antiviral drugs.

We previously described a novel isothermal nucleic acid amplification method, ‘Strand Invasion Based Amplification’ (SIBA®), with high analytical sensitivity and specificity (Hoser et al. 2014). The method relies on the recombinase-dependent insertion of a single-stranded invasion oligonucleotide (IO) into a complementary region of a target duplex DNA, which results in the dissociation of the target duplex. This dissociation event generates single-stranded DNA, which in turn allows target-specific primers to bind and extend the target sequence via the action of a DNA polymerase. The method was previously found to be useful for the rapid detection of DNA from pathogens (Hoser et al. 2014). In this study, we describe the development of a variant of SIBA technology, namely, reverse transcription SIBA (RT-SIBA), for the rapid detection of viral RNA targets. We demonstrate the efficacy of RT-SIBA in the detection of viral RNA by developing RT-SIBA assays for both influenza A and B. The assays were designed to detect sequences within conserved regions of the influenza genome. The RT-SIBA method includes a reverse transcriptase enzyme that allows a one-step reverse transcription of RNA to cDNA and simultaneous amplification and detection of the cDNA with SIBA under isothermal reaction conditions. Furthermore, we compared the performance of RT-SIBA with the previously published Centers for Disease Control and Prevention (CDC) real-time PCR protocol for the detection of influenza A and B.

## Materials and methods

### Oligonucleotides and sample material

**Fig. 1:**
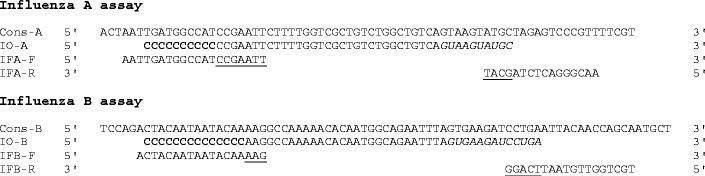
Alignment of oligonucleotides with consensus sequences. *Italic sequence* denotes 2′-O-methyl RNA sequence present in invasion oligonucleotides. *Bold poly-cytosine* denotes the non-homologous seeding area present in invasion oligonucleotides. *Underlined sequence* denotes the overlap between the forward or reverse primers and their respective invasion oligonucleotides

The oligonucleotides used in this study were purchased from Eurofins Genomics (Ebersberg, Germany) or Integrated DNA Technologies (Leuven, Belgium) and purified by the manufacturer using either reverse-phase HPLC (for primers and probes) or PAGE (for the IOs). Oligonucleotide sequences are shown in Fig. 1. Amplirun purified and quantified RNA from influenza A (H1N1, H3N2, and H5N1) and B were obtained from Vircell (Granada, Spain) and used to determine the analytical sensitivity of both the RT-SIBA and RT-qPCR methods. NATtrol FLU Verification Panel (ZeptoMetrix, Buffalo, NY, USA) samples were used to establish the ability of RT-SIBA to detect influenza viral particles in the presence of a simulated clinical sample matrix. Each pathogen was transferred using nasopharyngeal swabs into a lysis buffer (10 % Triton X-100, Sigma-Aldrich, USA) and 2 μl was then added to the reaction.

### Transcript and nucleic acid preparation

A 300-bp template sequence containing the region 2023–2322 from the influenza A virus segment 1 polymerase PB2 gene (KJ942711) was inserted into the pEXA2 vector by a commercial company (Eurofins Genomics, Ebersberg, Germany). The sequence was flanked by a 20-bp T7 promoter sequence and a *Sma*I site at the 5′ and 3′ ends, respectively. Transcripts were prepared using an in vitro transcription kit (HiScribe™ T7 High Yield RNA Synthesis Kit; New England Biolabs, Ipswich, MA, USA). Transcripts were quantified both spectrophotometrically and by quantitative real-time PCR and were used to determine the performance and analytical sensitivity of the influenza A RT-SIBA assay.

### RT-SIBA influenza A and B assay design

The Influenza Research Database (http://www.fludb.org) was used to aid the design of the influenza RT-SIBA assays (Squires et al. 2012). To design an assay for influenza A, a total of 4575 non-duplicate influenza A segment 1 sequences that had been submitted to the database during the years 2010–2015 were retrieved and aligned. Special emphasis was put on the most clinically relevant subtypes, H1N1, H3N2, and H5N1; thus, sequences representing these subtypes were included in the alignment. The alignment was analyzed to find areas of limited variability using Jalview (http://www.jalview.org). A highly conserved area of approximately 80 nucleotides was identified at the 3′ end of the aligned segment 1, with a suitable consensus sequence, and this area was selected as the target area for RT-SIBA assay design. Analogously, for the influenza B assay, an alignment of 1495 non-duplicate segment 3 sequences was produced. A highly conserved, approximately 80 nucleotide area was identified at the far 5′ end of the alignment and selected as the assay target area. Alignments of the consensus sequences for influenza A and B with the oligonucleotides designed for the SIBA influenza assays are shown in Fig. 1.

In essence, SIBA amplification is highly specific; therefore, special care was taken to ensure that the majority of any sequence variation fell inside the forward or reverse primers sequences and was limited to a maximum of 1–2 nucleotide changes in the IO region (see Hoser et al. (Hoser et al. 2014) with respect to general assay design, SIBA specificity, and initial screening of suitable oligo combinations). After the assays were established, they were subjected to several rounds of optimization as described below.

### RT-SIBA assays

RT-SIBA reactions were performed using a commercial SIBA reagent kit (Orion Diagnostica Oy, Espoo, Finland) with the addition of 16 U of GoScript™ Reverse Transcriptase (Promega, Madison, USA). UvsX and gp32 enzymes were each used at 0.25 mg/ml, and the reactions were started using 10 mM magnesium acetate. For the SIBA influenza A assay, the forward and reverse primers and the IO were each used at final concentrations of 400 nM. For the SIBA influenza B assay, the forward and reverse primers and the IO were each used at final concentrations of 200 nM. SIBA amplification was detected using SYBR Green 1 (dilution, 1:100,000; Thermo Fisher Scientific, USA). RT-SIBA reactions were incubated at 41 °C for 60 min, and fluorescence readings were taken at 60-s intervals on an Agilent MX Pro 3005P instrument (Agilent Technologies, Inc., CA, USA). After incubation for 60 min, the instrument was set to run a melt curve from 40 to 95 °C, to further assess the specificity of the SIBA reactions.

### Optimization of the influenza A and B RT-SIBA assays

SIBA reactions were optimized for the rapid detection of influenza A by first determining the best nucleotide configuration for the IO to give the shortest detection time. This was achieved by varying the length and composition of the 5′ non-homologous (seeding) region of the IO. Six different IOs with seeding regions of lengths varying from 0 to 17 nucleotides were tested. In another approach, the length of the seeding region was maintained at ten nucleotides but the nucleotide composition of the seeding region was modified to contain either polyC, polyT, polyA, polyG, poly purines, or poly pyrimidines. The exact sequences of each tested IO are listed in Table S1. The performance of each IO at 200 nM was assessed by its ability to facilitate amplification of 10,000 copies of in vitro transcribed influenza A RNA in the presence of the primers. The best performing seeding region was then chosen for subsequent SIBA influenza A experiments and adopted for influenza B SIBA assay design.

### Real-time RT-PCR

The performance of RT-SIBA assays for the detection of influenza A and B was compared with the CDC protocol for real-time RT-PCR of influenza A and B ((WHO) 30 April 2009; Selvaraju and Selvarangan 2010). Briefly, the RT-PCR reactions were performed with the EXPRESS One-Step SuperScript® qRT-PCR SuperMix Kit, with ROX Reference Dye (Thermo Fisher Scientific, USA). This kit contains a heat-labile form of uracil DNA glycosylase (UDG), in addition to SuperScript® III Reverse Transcriptase and Platinum® Taq DNA polymerase, in order to prevent any potential carry-over contamination. The reactions were detected using an Applied Biosystems 7500 PCR instrument (Thermo Fisher Scientific, USA). All primers and probes were used at 1 μM and 200 nM, respectively. The following thermal cycling protocol was used: 50 °C for 30 min (cDNA synthesis), 95 °C for 2 min (reverse transcription and UDG inactivation), and 45 cycles of 95 °C for 15 s and 55 °C for 30 s (PCR amplification).

## Results

### Assay optimization to minimize detection time

**Fig. 2:**
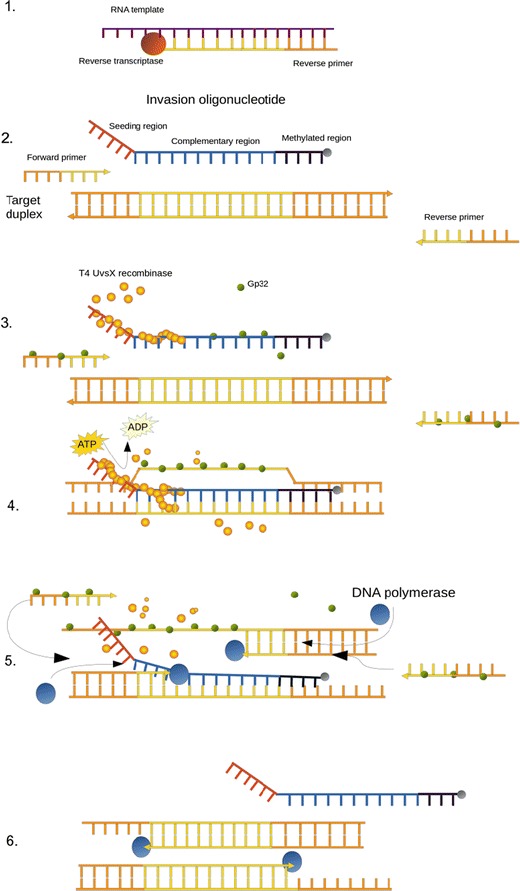
**a** Mechanistic description of the RT-SIBA reaction. *1* RNA is reverse transcribed to cDNA by the transcriptase enzyme. *2* The SIBA reaction requires two target-specific primers and an invasion oligonucleotide (IO). All single-stranded elements are coated with gp32. *3* T4 UvsX recombinase polymerizes the IO displacing bound gp32. The lengths of the primers used are too short to act as substrates for UvsX. *4* IO invades the complementary region of the target duplex through the activity of UvsX. The invasion process facilitates the complete separation of the target duplex, allowing target-specific primers to bind the target. *5* The strand displacement polymerase is able to extend the dissociated target duplex from the primers. This event leads to the production of two copies of the target duplex. The recombinase-mediated target duplex separation and polymerase-mediated extension are the basis for exponential amplification

**Fig. 3:**
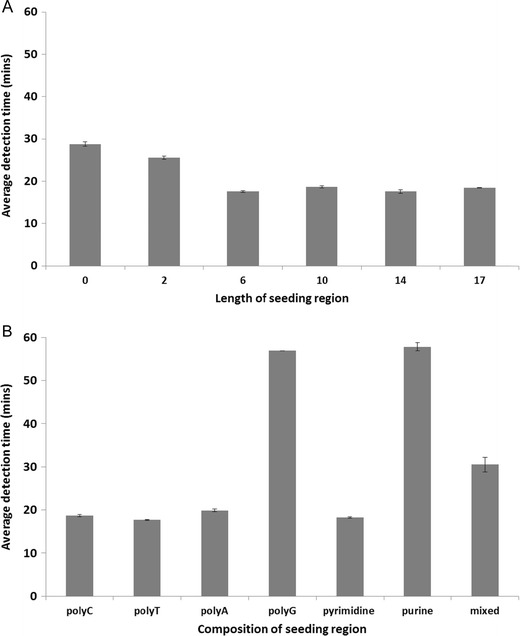
The impact of changes to the invasion oligonucleotide (IO) seeding region nucleotide composition on the average detection time for the SIBA influenza A assay. The impact of the seeding region **a** length and **b** composition (for a ten nucleotide seeding region). The results are presented as the average detection time from eight replicate assays (standard deviation included). The detection time reported was the time at which the probe fluorescent signal exceeded the background signal

The mechanism of RT-SIBA is described in Fig. 2. The IO, used in SIBA reactions to separate the target duplex DNA, includes a 5′ region that is not homologous to the target sequence. This non-homologous region, termed the seeding region, is included in the IO to allow for optimal coating of the homologous portion by the recombinase. The affinity of the recombinase for DNA molecules was previously suggested to be dependent on both the length and nucleotide composition of the target molecule (Formosa and Alberts 1986). Therefore, we hypothesized that the length and composition of the seeding region could also impact the speed of SIBA assays. In order to test our hypothesis, SIBA influenza A assay IOs containing seeding regions that differed in length and nucleotide composition were designed and tested, and the results are depicted in Fig. 3. The use of an IO with no seeding region resulted in reactions with the longest detection time, suggesting that the seeding region is of vital importance for efficient amplification of the target DNA. Furthermore, shorter detection times were observed as the length of the seeding region increased. Our experiments demonstrate that the optimal length of the seeding region, in terms of assay speed, is a minimum of six nucleotides. To evaluate the impact of the nucleotide composition of the seeding region on assay speed, seven IOs with seeding regions containing either polyC, polyT, polyA, polyG, poly purines, poly pyrimidines, or mixed nucleotides were tested. The use of IO seeding regions containing polyC and polyT resulted in the shortest detection times, while the use of an IO containing a polyG seeding region led to the longest detection time. The IO containing a seeding region with a mixture of pyrimidine nucleotides was found to display shorter detection times than that with a mixture of purine nucleotides. In conclusion, IOs for both influenza A and B assays used in the following experiments were designed to contain seeding regions consisting of a polyC sequence of 10 and 14 nucleotides in length, respectively.

### Sensitivity and inclusivity of influenza A and B RT-SIBA assays in comparison with RT-PCR

**Fig. 4:**
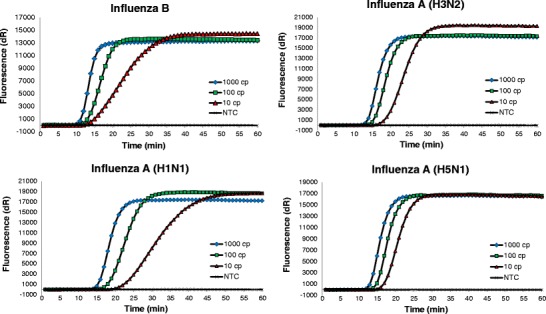
Sensitivity and inclusivity of influenza A and B RT-SIBA assays. Serial dilutions of RNA from different influenza serotypes were prepared and used as templates in RT-SIBA assays. The reactions were run in quadruplicate in three individual experiments. The figures show data from one experiment, and the data are plotted as the average of four replicate reactions. The total copy number of template RNA per reaction was 10–1000 copies/reaction. The results indicated that RT-SIBA could be used to reproducibly detect as few as ten copies of influenza RNA per reaction. NTC = no template control

The sensitivities of both the influenza A and B RT-SIBA assays were assessed against those of the equivalent previously published CDC real-time RT-PCR assays. Serial dilutions of quantified Amplirun RNA controls for influenza A H1N1, H3N2, and H5N1 subtypes, as well as influenza B, were used as templates for both RT-SIBA and RT-PCR assays. Quadruplicate reactions were performed in at least three independent experiments. The diluted samples were analyzed simultaneously with both RT-SIBA and RT-PCR reactions in order to facilitate comparison between the two methods. The results are shown in Fig. 4 and Table 1.

**Table 1:** Sensitivity of the influenza A and B RT-SIBA assays, compared with equivalent CDC RT-PCR assays

		No. of positive reactions (average time to positive result*)			
		RT-SIBA		RT-PCR	
Subtype	Copies/reaction	Influenza A	Influenza B	CDC-A	CDC-B
A (H1N1)	1000	12/12 (12 min)		12/12 (54 min)	
	100	12/12 (15 min)		0/12	
	10	12/12 (20 min)		0/12	
A (H3N2)	1000	12/12 (11 min)		12/12 (50 min)	
	100	12/12 (14 min)		12/12 (52 min)	
	10	12/12 (16 min)		0/12	
A (H5N1)	1000	12/12 (10 min)		12/12 (51 min)	
	100	12/12 (12 min)		12/12 (53 min)	
	10	12/12 (15 min)		0/12	
B	1000		12/12 (10 min)		12/12 (51 min)
	100		12/12 (12 min)		12/12 (53 min)
	10		11/12 (13 min)		11/12 (56 min)

*Ramp time of RT-PCR reactions not included in the calculations

RT-SIBA reliably detected as low as ten copies of viral RNA per reaction. Serial dilutions from 1000 to 10 copies of purified RNA from different influenza subtypes showed reproducible and specific amplification when detected using SYBR green I fluorescent dye. Post-amplification melt curve analysis was performed after each run, and amplification of a single-specific amplicon was detected for both influenza A and B assays (data not shown). Furthermore, the influenza A RT-SIBA assay did not amplify influenza B RNA or vice versa.

Comparison of analytical sensitivity in terms of RNA copy numbers per reaction indicated that the RT-SIBA assays had comparable or superior sensitivity, compared with RT-PCR assays (Table 1). Notably, we were able to demonstrate the sensitivity of RT-SIBA to be ten copies of target RNA per reaction for both influenza A and B assays; however, our experiments demonstrated that, unlike that of the RT-SIBA assays, the sensitivity of RT-PCR varied depending on the assay and influenza template subtype used. For influenza B assays, RT-SIBA and RT-PCR assays performed equally well and were both able to successfully amplify ten copies of RNA per reaction. However, a significant difference between RT-SIBA and RT-PCR was observed for the influenza A assays. Ten copies of all tested subtypes of influenza A were reproducibly amplified and detected by RT-SIBA, whereas the RT-PCR assay was only capable of amplifying 100 or 1000 copies of RNA per reaction.

The average time to achievement of a positive reaction, corresponding to the threshold cycle (Ct)-value for RT-PCR reactions, was calculated according to the one-step RT-PCR cycling program, without taking into account the ramping time needed for PCR cycling. RT-SIBA reactions were performed at a constant temperature, and data were collected at 1 min intervals. Therefore, the Ct-values of RT-SIBA reactions corresponded directly to the time required to achieve a positive result. The average times to positive results for both RT-SIBA and RT-PCR reactions are listed in Table 1.

Positive results from 100 copies of influenza B RNA template per reaction were obtained in an average of 12 min with the RT-SIBA assay, whereas an average of 53 min was required to yield a positive result by RT-PCR. Similarly, 100 copies of influenza A H1N1, H3N2, and H5N1 subtypes were detected by RT-SIBA in 15, 14, and 12 min, respectively, whereas reaction times of more than 50 min were needed for RT-PCR. These results demonstrate a major advantage of RT-SIBA over RT-PCR.

### Analytical specificity of RT-SIBA influenza A and B assays and their performance in analysis of a clinical panel

The specificities of the SIBA influenza A and B assays were elucidated by challenging the reactions with nucleic acid from six other common human respiratory pathogens. These included human rhinovirus, respiratory syncytial virus (RSV), coronavirus, adenovirus, *staphylococcus aureus*, and *streptococcus pyogenes*. All non-influenza nucleic acid templates gave negative results with both the influenza A and B RT-SIBA assays (data not shown). The influenza A assay was further challenged with an influenza B RNA template, which was not detected by the assay. Similarly, the influenza B assay did not detect influenza A template RNA. These findings suggest that both of the SIBA influenza assays are specific for their respective targets.

The performance of both the influenza A and B RT-SIBA assays was further evaluated using commercial NATtrol FLU verification panel samples that contain viral or bacterial particles in a protein matrix, to mimic clinical specimen samples. The panel consisted of six influenza A virus H1 and H3 strains, two influenza B strains, respiratory syncytial virus A, respiratory syncytial virus B, rhinovirus 1 A, parainfluenza virus type 1, Echovirus Type 30, Coxsackievirus type A9, *Mycoplasma pneumoniae* strain M129, and *Neisseria meningitidis* Serogroup A. The results are shown in Table 2. The influenza A RT-SIBA assay detected all six influenza A strains and did not cross-react with influenza B strains, nor the other respiratory pathogens. Similarly, the influenza B RT-SIBA assay detected the two influenza B strains and did not cross-react with influenza A strains, nor the other respiratory pathogens. This indicates that the influenza A and B RT-SIBA assays are likely to be suitable for detecting influenza viruses from clinical specimens.

**Table 2:** NATtrol FLU verification panel test results

Panel Member	Strain	SIBA result*,* Influenza A	SIBA result*,* Influenza B
Influenza A H1N1	A/NewCaledonia/20/99	19.4	–
Influenza A H1N1	A/Brisbane/59/07	20.1	–
Influenza A H3N2	A/Brisbane/10/07	17.4	–
Influenza A H3N2	A/Wisconsin/67/05	18.4	–
Influenza A 2009 H1N1	Canada/6294/09	18.6	–
Influenza A 2009 H1N1	NY/02/09	19.9	–
Influenza B	B/Florida/02/06	–	14.8
Influenza B	B/Malaysia/2506/04	–	18.1
Respiratory syncytial virus A	NA	–	–
Respiratory syncytial virus B	CH93(18)-18	–	–
Rhinovirus 1 A	NA	–	–
Parainfluenza virus type 1	NA	–	–
Echovirus Type 30	NA	–	–
Coxsackievirus type A9	NA	–	–
*M. pneumoniae*	M129	–	–
*N. meningitidis* Serogroup A	NA	–	–

Time to detection (in minutes) of positive reactions is shown in the table. Data represent averages of five replicates from one representative run. The number of viral particles per sample was not reported by the manufacturer

## Discussion

In this study, we demonstrated the ability of SIBA technology to detect RNA-pathogens, RT-SIBA, for the rapid detection of influenza A and B with high analytical sensitivity and specificity. Previously, it was demonstrated that SIBA technology is a feasible method for the reliable detection of DNA from pathogenic micro-organisms (Hoser et al. 2014). In this paper, we demonstrate that RT-SIBA can be applied for the rapid detection of pathogens that have RNA, rather than DNA, as their genetic material, as is the case for the majority of viruses. The method described here includes a reverse transcriptase enzyme in the same reaction tubes as the other SIBA reaction components required for nucleic acid amplification and consequently permits a one-step reverse transcription of RNA to cDNA and simultaneous amplification and real-time detection of cDNA by SIBA.

The SIBA method consists of a recombinase, required for homologous recombination, an IO, which is a substrate for the recombinase, and two target-specific primers that are not substrates for the recombinase. The IO separates the target duplex, with the aid of the recombinase, consequently allowing the primers to bind and extend the target via the action of a DNA polymerase. Repeated cycles of duplex separation and primer extension are the basis for exponential amplification. In this report, the amplification times for the SIBA influenza assays were first optimized by varying the length and composition of the IO non-homologous region (i.e., the seeding region). Our experiments showed that a polyC or polyT non-homologous IO region of at least ten nucleotides in length resulted in the shortest detection times. The recombinase, UvsX binds preferentially to pyrimidine rich nucleotides (Formosa and Alberts 1986), however, the reaction demands both the binding and release of the recombinase and consequently the optimal configuration of the seeding region was determined empirically. The necessity of the seeding region demonstrated that the recombinase perform less efficiently at the initial potential binding sites prior to its polymerization from 5′-3′.

The average times to detection for both the influenza A and B RT-SIBA assays were below 15 min for 100 copies of RNA. Thus, the complete test can be conducted in less than 30 min. This is significantly faster than the real-time RT-PCR method, which took over 1 h for the detection of influenza. In RT-SIBA, the reverse transcription and cDNA amplification occur simultaneously and at the same reaction temperature, thus facilitating rapid detection of target RNA. By contrast, in real-time RT-PCR, reverse transcription requires an initial incubation period of 30 min at a constant temperature prior to the repeated PCR cycles of denaturation, annealing, and elongation, resulting in a total run time of 90–120 min. Furthermore, RT-SIBA was found to be more sensitive than RT-PCR for the detection of influenza A and B. RT-SIBA displayed a 100-fold improvement in sensitivity, compared with RT-PCR, for the detection for H1N1. The SIBA reaction components include the single strand binding protein, gp32, which acts as a recombinase accessory protein and is required for the removal of secondary structures within the oligonucleotides used in the assay. It is possible that the presence of gp32 in the RT-SIBA reaction mixture could also contribute to the high analytical sensitivity displayed by RT-SIBA for RNA targets. This is supported by previous studies, which showed that single binding proteins enhance the yield of cDNA synthesis by reverse transcriptase, thus improving DNA amplification sensitivity (Jefferies and Farquharson 2002; Villalva et al. 2001). Furthermore, the difference in RT enzymes used in RT-SIBA and RT-PCR could account for the significant difference in sensitivity between the methods. Several studies show that commercially available reverse transcriptases display varying performance regarding cDNA yield and sensitivity (Levesque-Sergerie et al. 2007; Okello et al. 2010; Ståhlberg et al. 2004).

The performance of RT-SIBA for the detection of influenza A and B was determined using the commercially available clinical NATtrol FLU verification panel samples, which simulate clinical specimen samples. The RT-SIBA influenza assays amplified the influenza specimens specifically, with no cross-reactions with other respiratory pathogens. Since RT-SIBA was found to be less susceptible to sample derived inhibition, the method does not require highly purified RNA template material and crude disruption of viral particles using detergents was found to be sufficient. The sensitive and rapid detection of influenza viruses within 15 min, as well as the tolerance to sample derived inhibition, demonstrate that these assays are powerful molecular diagnostics tools. Since the method is performed at a low and constant temperature, there is potential for it to be run using battery operated and portable devices of considerably lower complexity than those used for RT-PCR. Isothermal methods can reasonably be expected to extend the use of molecular methods outside of centralized laboratory spaces into lower complexity settings, such as smaller laboratories, the field, or even domestic homes.

## Electronic supplementary material

ESM 1 (PDF 169 kb)

## References

[CR1] Formosa T, Alberts BM (1986). Purification and characterization of the T4 bacteriophage uvsX protein. J Biol Chem.

[CR2] Gerard GF, Potter RJ, Smith MD, Rosenthal K, Dhariwal G, Lee J, Chatterjee DK (2002). The role of template-primer in protection of reverse transcriptase from thermal inactivation. Nucleic Acids Res.

[CR3] Hoser MJ, Mansukoski HK, Morrical SW, Eboigbodin KE (2014). Strand invasion based amplification (SIBA®): a novel isothermal DNA amplification technology demonstrating high specificity and sensitivity for a single molecule of target analyte. PLoS one.

[CR4] Jefferies D, Farquharson C (2002). Effects of choice of reverse-transcriptase enzyme and use of T4 gene 32 protein on banding patterns in agarose gel differential display. Anal Biochem.

[CR5] Levesque-Sergerie J-P, Duquette M, Thibault C, Delbecchi L, Bissonnette N (2007). Detection limits of several commercial reverse transcriptase enzymes: impact on the low- and high-abundance transcript levels assessed by quantitative RT-PCR. BMC Mol Biol.

[CR6] Low D (2008). Reducing antibiotic use in influenza: challenges and rewards. Clin Microbiol Infect.

[CR7] Mahony JB, Petrich A, Smieja M (2011). Molecular diagnosis of respiratory virus infections. Crit Rev Clin Lab Sci.

[CR8] Mallia P, Johnston SL (2007). Influenza infection and COPD. Int J Chron Obstructive Pulm Dis.

[CR9] Okello JBA, Rodriguez L, Poinar D, Bos K, Okwi AL, Bimenya GS, Sewankambo NK, Henry KR, Kuch M, Poinar HN (2010). Quantitative assessment of the sensitivity of various commercial reverse transcriptases based on armored HIV RNA. PLoS ONE.

[CR10] Selvaraju SB, Selvarangan R (2010). Evaluation of three Influenza A and B real-time reverse transcription-PCR assays and a new 2009 H1N1 assay for detection of Influenza viruses. J Clin Microbiol.

[CR11] Simonsen L, Fukuda K, Schonberger LB, Cox NJ (2000). The impact of Influenza epidemics on hospitalizations. J Infect Dis.

[CR12] Squires RB, Noronha J, Hunt V, García-Sastre A, Macken C, Baumgarth N, Suarez D, Pickett BE, Zhang Y, Larsen CN, Ramsey A, Zhou L, Zaremba S, Kumar S, Deitrich J, Klem E, Scheuermann RH (2012). Influenza research database: an integrated bioinformatics resource for influenza research and surveillance. Influenza Other Respir Viruses.

[CR13] Ståhlberg A, Kubista M, Pfaffl M (2004). Comparison of reverse transcriptases in gene expression analysis. Clin Chem.

[CR14] Stiver G (2003). The treatment of influenza with antiviral drugs. CMAJ: Canadian Medical Association Journal.

[CR15] Villalva C, Touriol C, Seurat P, Trempat P, Delsol G, Brousset P (2001). Increased yield of PCR products by addition of T4 gene 32 protein to the SMART PCR cDNA synthesis system. Biotechniques.

[CR16] World Health Organisation (WHO) (2009) CDC protocol of real-time RT-PCR for influenza H1N1. World Health Organization, Geneva. http://www.who.int/csr/resources/publications/swineflu/realtimeptpcr/en/index.html

