# Supplementary material for: Reverse transcription strand invasion based amplification (RT-SIBA): a method for rapid detection of influenza A and B
Source: Appl Microbiol Biotechnol. 2016 Apr 11;100(12):5559–67. doi: 10.1007/s00253-016-7491-y (PMC4875950; doi:10.1007/s00253-016-7491-y)
Supplement: Supplementary file 1 — (PDF 169 kb) [file 253_2016_7491_MOESM1_ESM.pdf]

Applied Microbiology and Biotechnology

Reverse Transcription Strand Invasion Based Amplification (RT-SIBA): A Method for Rapid Detection of Influenza A and B

Supplementary data

Kevin Eboigbodin[1,#], Sanna Filén[1,*], Tuomas Ojalehto[1,*], Mirko Brummer[1], Sonja Elf[1], Kirsi Pousi[1], and Mark Hoser[2]

[1]Research and Development, Orion Diagnostica, Oy, P. O. BOX 83, FI-02101 Espoo, Finland

[2] Molecular Biology, GeneForm Technologies, Broadstairs, United Kingdom

*Authors with equal contribution

[#]corresponding author

To whom correspondence should be addressed: Kevin E. Eboigbodin, Research and Development, Orion Diagnostica Oy, P.O.Box 83, FI-02101, Espoo, Finland,

Tel:+358509665879, E-mail: kevin.eboigbodin@oriondiagnostica.fi

Table S1 – Invasion oligonucleotides (IO) used for optimisation of SIBA influenza A assay

SIBA Influenza A IO	Sequence
0	CCGAATTCTTTTGGTCGCTGTCTGGCTGTCAmGmUmAmAmGmUmAmUmGmC
poly 2C	**CC** CCGAATTCTTTTGGTCGCTGTCTGGCTGTCAmGmUmAmAmGmUmAmUmGmC
poly 6C	**CCCCCC** CCGAATTCTTTTGGTCGCTGTCTGGCTGTCAmGmUmAmAmGmUmAmUmGmC
poly 10C*	**CCCCCCCCCC** CCGAATTCTTTTGGTCGCTGTCTGGCTGTCAmGmUmAmAmGmUmAmUmGmC
poly 14C	**CCCCCCCCCCCCCC** CCGAATTCTTTTGGTCGCTGTCTGGCTGTCAmGmUmAmAmGmUmAmUmGmC
poly 17C	**CCCCCCCCCCCCCCCCC** CCGAATTCTTTTGGTCGCTGTCTGGCTGTCAmGmUmAmAmGmUmA
polyT	**TTTTTTTTTT** CCGAATTCTTTTGGTCGCTGTCTGGCTGTCAmGmUmAmAmGmUmAmUmGmC
polyA	**AAAAAAAAAA** CCGAATTCTTTTGGTCGCTGTCTGGCTGTCAmGmUmAmAmGmUmAmUmGmC
polyG	**GGGGGGGGGG** CCGAATTCTTTTGGTCGCTGTCTGGCTGTCAmGmUmAmAmGmUmAmUmGmC
pyrimidine	**TCTCTCTCTC** CCGAATTCTTTTGGTCGCTGTCTGGCTGTCAmGmUmAmAmGmUmAmUmGmC
purine	**AGAGAGAGAG** CCGAATTCTTTTGGTCGCTGTCTGGCTGTCAmGmUmAmAmGmUmAmUmGmC
mixed	**CTAGATCTGA** CCGAATTCTTTTGGTCGCTGTCTGGCTGTCAmGmUmAmAmGmUmAmUmGmC

In invasion oligonucleotides bold sequences denote non-homologous seeding area sequence. *mA*, *mC*, *mG* and *mU* denote 2'-O-methyl RNA nucleotides. *poly 10C was used for subsequent SIBA influenza A experiments
